# Adrenomedullin in peritoneal effluent expressed by peritoneal mesothelial cells

**DOI:** 10.1007/s10157-013-0801-0

**Published:** 2013-04-06

**Authors:** Rika Kono, Koichi Kanozawa, Tatsuo Shimosawa, Yousuke Tayama, Akihiko Matsuda, Hajime Hasegawa, Tetsuya Mitarai

**Affiliations:** 1 Division of Nephrology and Hypertension, Saitama Medical Center, Saitama Medical University, 1981 Kamoda, Kawagoe, Saitama 350-8550 Japan; 2 Department of Clinical Laboratory and JST CREST, The University of Tokyo School of Medicine, Tokyo, Japan

**Keywords:** Adrenomedullin, Peritoneal dialysis, Peritoneal mesothelial cells, Amidation

## Abstract

**Background:**

Adrenomedullin (AM) possesses vasodilative and cell-protective properties. Glycine combines with the C-terminal of AM to form mature, physiologically active AM (mAM). AM is reportedly induced by high glucose condition in vascular endothelial or smooth muscle cells; however, little is known on how AM is activated by amidation. To investigate the behavior of AM in patients undergoing peritoneal dialysis (PD), the concentrations of AM, mAM and CA125 were measured. The mAM to AM ratio (mAM/AM ratio) was also evaluated as a marker of amidation activity.

**Methods:**

Twenty patients were recruited for this study. The effluent at the time of the peritoneal equilibration test was collected and AM, mAM and CA125 concentrations were measured. The expression of AM in peritoneal mesothelial cells (PMCs) collected from effluent was also examined with an indirect immunofluorescent method.

**Results:**

Mean values of AM and mAM in effluent were 18.1 ± 1.6 and 4.1 ± 0.3 fmol/mL, respectively. In plasma, they were 42.6 ± 3.3 and 5.6 ± 0.6 fmol/mL, respectively. AM concentrations in effluent did not correlate with plasma AM level but correlated well with the dialysate-to-plasma ratio of creatinine (D/P ratio of creatinine). Moreover, in 7 of 20 cases, concentrations of the mAM and mAM/AM ratio in effluent were higher than in plasma. In effluent, AM concentration but not the mAM/AM ratio correlated with CA125 concentration. Immunocytological study revealed diffuse, cytoplasmic expression of AM in PMCs which were collected from effluent during PD.

**Conclusion:**

AM is expressed by PMCs and actively amidated in the abdominal cavity of patients undergoing PD.

## Introduction

Adrenomedullin (AM) is comprised of 52 amino acids and was originally isolated in pheochromocytoma tissue by its ability to elevate cAMP in rat platelets. It is now recognized as a potent circulating vasodilatory peptide which is secreted by ubiquitous cells and organs [1]. Because the cytoprotective effect of AM is mediated by the cAMP signaling pathway, it is expected that AM is involved in various cellular processes [2]. Circulating AM is mainly secreted from vascular endothelial and smooth muscle cells. AM is processed from its precursor as the intermediate form. Subsequently, the intermediate form is converted by enzymatic amidation [3] to the biologically active mature form of AM (mAM).

Since AM is biologically active only after C-terminal amidation of immature AM, it is necessary to determine the level of mAM in order to investigate the pathological role of AM [4].

It has also been reported that hyperglycemia enhances AM expression in the vessels, indicating that AM is involved in the regulation of glycemic control [5]. Plasma AM concentration in diabetic patients is closely associated with diabetic vascular complications [6]. However, only limited information on mAM level or amidation activity is available. Generally, the dialysate used in peritoneal dialysis (PD) has a high glucose concentration of 1.5–2.5 %; this high glucose concentration leads to deterioration of the peritoneum.

The peritoneum of long-term PD patients was characterized by a loss of ultrafiltration capacity associated morphologically with loss of peritoneal mesothelial cells (PMCs), submesothelial fibrosis and neoangiogenesis, which were attributed to the high glucose condition [7]. Hence, high glucose condition in PD dialysate may stimulate AM expression and AM may play a role in the peritoneal status and serve as an indicator of PD patients. The peritoneum is composed not only of PMCs but also endothelial cells, fibroblasts and adipocytes. However, AM expression has not been confirmed in PMCs, which are a major constituent of the peritoneum. In this study of PD patients, AM and mAM levels were compared with the level of CA125, a bulk marker for the mesothelial mass [8], as well as evaluating amidation activity.

## Methods

### Patients

Twenty patients (male:female 12:8) treated with PD were enrolled in this study after obtaining informed consent (Table 1). The protocol was approved by the Ethics Review Board of Saitama Medical Center, Saitama Medical University. Heart failure (volume overload) was excluded. Patients were maintained on PD with exchange volumes of 1,500 or 2,000 mL and with at least four exchanges per day. Glucose concentrations of dialysate ranged from 1,350 to 2,272 mg/dL (average 1,611 mg/dL). Icodextrin-based dialysate and pH-neutral peritoneal dialysate were not used. In the present study we used the peritoneal equilibration test (PET) which was devised by Twardowski [9] as a grasp method for examination of peritoneal permeability. Standardized PET was performed on all patients by using the dialysate which had glucose concentrations of 2,270 or 2,500 mg/dL. The dialysate-to-instilled ratio of glucose (D4/D0 ratio of glucose) and the D/P ratio of creatinine were calculated from the data of PET. Effluent and plasma samples were collected from patients at the end-point of PET.

**Table 1:** Clinical features of patients

Number (male:female)	20 (12:8)
Age (years)	55 ± 2
Underlying renal disease	
Chronic glomerulonephritis	10
Diabetic nephropathy	2
Other/unknown	8
Peritoneum dialysis period (years)	4.7 ± 0.7
History of peritonitis (times)	0.4 ± 0.2 (0–2)
Concentration of glucose in peritoneal dialysis effluent (mg/dL)	1,611 ± 68

Data are expressed as the mean ± SE

### Measurement of AM, mAM, CA125, glucose, and creatinine concentration

Concentrations of AM and mAM in samples from effluent and plasma were measured by a one-step two-site immunoradiometric assay (IRMA) method using monoclonal antibodies (Cosmic Corporation, Tokyo, Japan). In addition, the mAM/AM ratio was calculated. Serum and effluent CA125 were measured by enzyme immunoassay (EIA) (Tosho Corporation, Tokyo, Japan). Serum and effluent glucose were measured by hexokinase and glucose-6-phosphate dehydrogenase methods. Serum and effluent creatinine levels were measured enzymatically (Mizuho Medy, Saga, Japan). Finally, the concentrations of AM, mAM and CA125 in effluent were examined for their relevance in a disease process such as diabetes.

### AM expression of PMCs in effluent

PMCs were collected in effluent by a cytocentrifuge, and immunocytological studies were performed using anti-human AM antibody, combined with anti-vimentin antibody. Four hundred milliliters of effluent were collected at the end-point of PET. Effluents were centrifuged for 10 min (1,500 rpm, 4 °C), and the pellet was suspended into a small amount of medium, then smears were made by cytospin preparations (800 cpm, 25 °C, 5 min). Specimens on slides were fixed in 3.7 % formalin for 10 min and briefly immersed (5 min) in 0.5 % TritonX-100. The slides were first incubated with rabbit anti-human AM antibody, followed by rhodamine-conjugated goat anti-rabbit IgG (1:100 dilution; Chemicon International, Inc., Temecula, CA, USA) as the second antibody. In order to identify PMCs, the slides were also incubated with mouse anti-vimentin antibody (PROGEN Biotechnik GmbH, Heidelberg, Germany). Then mouse IgG was detected by FITC-conjugated goat *F*(*ab*′) 2 anti-mouse immunoglobulin (1:100 dilution; Biosource International, Camarillo, CA, USA).

PMCs were identified by cell shape and positive staining of vimentin. Fluorescence intensity of rhodamine-labeled anti-AM antibodies in the cytoplasm was evaluated using laser scanning confocal microscopy (MRC-1000; Bio-Rad) under the following conditions (laser 30 %, iris 2.0 mm, gain 1,200 V), and average fluorescence intensity of rhodamine was calculated.

### Statistical analysis

All values were statistically analyzed by Student’s *t* test, and the *z* analysis was applied for % changes. *p* values <0.05 were considered significant.

## Results

**Fig. 1:**
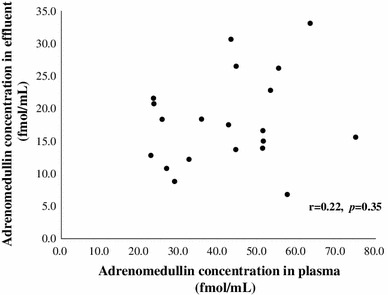
Lack of correlation between AM concentration in plasma and effluent

**Fig. 2:**
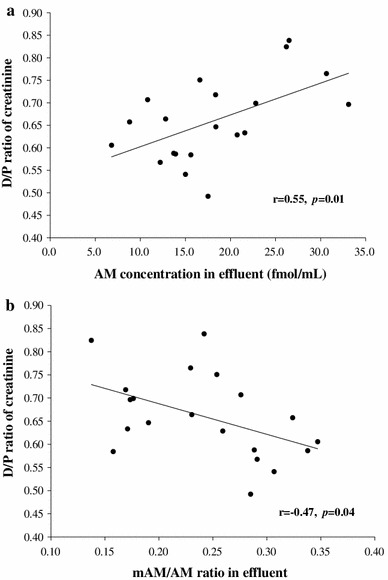
**a** A correlation between AM concentration in effluent and the D/P ratio of creatinine. **b**. A negative correlation between the mAM/AM ratio in effluent and the D/P ratio of creatinine

The characteristics of enrolled patients are summarized in Table 1. The average age of patients was 55 ± 2 years. Mean PD period was 4.7 ± 0.7 years. Table 2 shows the mean value of AM in effluent was significantly lower than in plasma. However, there was no correlation between AM concentration in plasma and in effluent (*p* = 0.35) (Fig. 1). The mAM/AM ratio in effluent was elevated to 0.242 ± 0.014 as compared with 0.130 ± 0.008 in plasma (*p* < 0.01). It was suggested that amidation was accelerated in the peritoneal cavity. There was no patient whose AM concentration in effluent was higher than in plasma. However, for mAM concentration, there were seven patients with higher values in effluent than in plasma. AM concentration in effluent correlated well with the D/P ratios of creatinine (*r* = 0.55, *p* = 0.01) (Fig. 2a), but not with the D4/D0 ratios of glucose (*r* = −0.40, *p* = 0.08). In contrast, mAM concentration in effluent did not correlate with either the D/P ratio of creatinine or the D4/D0 ratio of glucose. The mAM/AM ratio in effluent correlated with the D/P ratio of creatinine (*r* = −0.47, *p* = 0.04) (Fig. 2b) but not with the D4/D0 ratio of glucose. AM concentration in effluent did not correlate with the PD period (*p* = 0.88).

**Table 2:** Laboratory findings

	Plasma	Effluent	*p* value
Mean value of AM (fmol/mL)	42.6 ± 3.3	18.1 ± 1.6	<0.01
Mean value of mAM (fmol/mL)	5.6 ± 0.6	4.1 ± 0.3	<0.05
mAM to AM ratio	0.130 ± 0.008	0.242 ± 0.014	<0.01
Effluent concentration to plasma concentration ratio of AM			0.47 ± 0.05
Effluent concentration to plasma concentration ratio of mAM			0.85 ± 0.07

### AM, mAM concentration, mAM/AM ratio and CA125 concentration in effluent

**Fig. 3:**
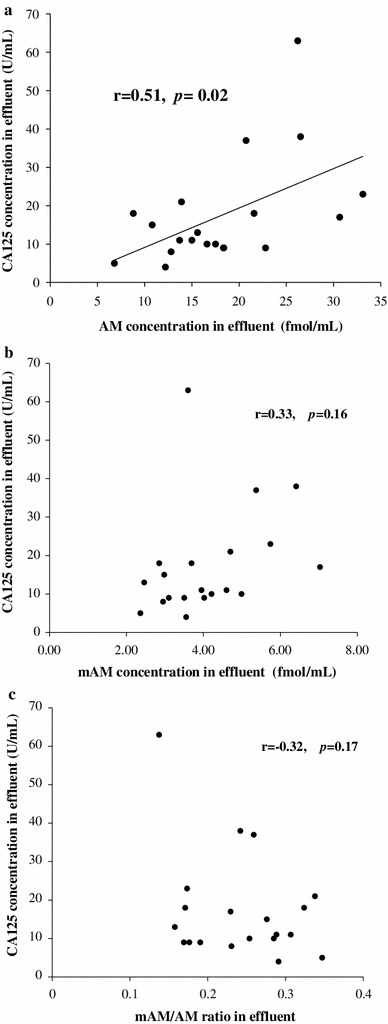
**a** A positive correlation between AM concentration in effluent and CA125 concentration in effluent. **b** A lack of correlation between mAM concentration in effluent and CA125 concentration in effluent. **c** A lack of correlation between the mAM/AM ratio in effluent and CA125 concentration in effluent

AM and CA125 concentrations in effluent showed positive correlation (*r* = 0.51, *p* = 0.02) (Fig. 3a). However, mAM and CA125 concentrations in effluent showed no correlation (*r* = 0.33, *p* = 0.16) (Fig. 3b). Similarly, the mAM/AM ratio and CA125 concentration in effluent showed no correlation (*r* = −0.32, *p* = 0.17) (Fig. 3c).

### AM expression of PMCs in effluent

**Fig. 4:**
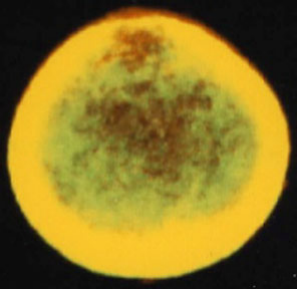
A representative example of PMCs showing diffuse expression of AM in the cytoplasm. Expression of AM was confirmed by double staining. Rhodamine showed expression of AM, and FITC-stained vimentin. The cytoplasmic portion with AM is shown in *red*. The overlap of AM and vimentin is shown in *yellow*

Immunocytological study revealed that AM was diffusely expressed in the cytoplasm of PMCs. A representative example of PMCs producing AM is shown in Fig. 4. Rhodamine fluorescence, measured semi-quantitatively by confocal laser microscopy, was not detected in the vimentin-negative cells. The fluorescence intensity using confocal laser microscopy for the anti-AM antibody on the cells identified as PMCs had a standard deviation 558 ± 142-fold stronger signal than the cells which were vimentin-negative. The absence of AM indicated the cells were not PMCs. On the other hand, the vimentin-positive cells could be used to calculate the intensity of rhodamine.

## Discussion

AM was isolated from the adrenal medulla and is a potent vasodilative peptide [1]. mRNA of human AM is highly expressed in pheochromocytoma as well as in various tissues or cells, including normal adrenal medulla, kidney, lung, and heart [10]. AM levels in plasma of patients with poorly controlled diabetes were significantly higher than in healthy volunteers. This suggests that the elevated plasma levels of AM may originate from vascular AM exposed to hyperglycemia via protein kinase C-dependent pathway [5, 11]. Post-translational amidation turns AM into its active form, mAM [1], but precise mechanisms of amidation or an enzyme responsible for amidation has not been identified.

In PD therapy, PMCs are exposed to high glucose by dialysate and they may express AM. In addition to endothelial cells, fibroblasts and adipocytes, the present study is the first to show that AM is also expressed by PMCs. Immunocytological study revealed that AM was diffusely expressed in the cytoplasm of PMCs of PD patients. As AM is a cytoprotective peptide and is upregulated by high glucose condition, the expression of AM in PMCs during PD might contribute to protect PMCs.

Using the same assay as in this study, it was reported that plasma AM and mAM concentrations in healthy individuals were 2.80 ± 0.14 and 0.65 ± 0.06 fmol/mL, respectively [12]. Another report showed that the mean plasma AM concentration was higher in pre-hemodialysis patients than in healthy volunteers [13]. Additionally, we reported that mAM concentrations in plasma of hemodialysis patients at the beginning and end of the hemodialysis treatment were 3.0 ± 0.3 and 2.8 ± 0.2 fmol/mL, respectively [14]. These values are higher than in healthy subjects [12].

Although absolute values of mAM and AM were low in effluent, the mAM/AM ratio was higher in effluent than in plasma, suggesting a higher amidation activity [15]. An amidation enzyme for AM has not been identified but it is possible that amidation is increased in the abdominal cavity of PD patients than in the plasma by high glucose condition. Further study will be necessary to clarify the regulation of amidation activity by glucose.

AM level in effluent correlated with CA125, a marker for PMCs number, and immunocytochemistry showed that PMCs in effluent express AM. However, the mAM/AM ratio did not correlate with CA125. This suggests that injured PMCs possess only low amidation activity. The mAM/AM ratio negatively correlated with the D/P ratio of creatinine, suggesting that injured peritoneum can amidate AM. Clearly further study is required to identify the cells responsible for amidating AM.

The molecular weight of AM is 6,028 Kd and it is conceivable for AM to penetrate the peritoneum [16]. In the present study, AM in effluent correlated with the D/P ratio of creatinine (Fig. 2a). Thus, AM level should be higher than in plasma of patients with deteriorating peritoneal function. However, the AM concentrations in effluent and plasma were not correlated and were even lower than in plasma. AM in effluent is the sum of locally expressed AM and dialyzed AM from blood, and is actively amidated and degradated. Furthermore, AM is diluted by dialysate. We showed that detached PMCs in effluent store AM and that AM level in effluent is correlated with CA125. Taken together, it suggests that AM in effluent might be leakage from injured PMCs, and AM from injured PMCs constitute most of the AM in effluent.

The peritoneum was not obtained in this study. Therefore, we could not fully elucidate the organ-protective effect of AM or clinical implications of AM in PD patients. The cells that express and amidate AM in the peritoneum were not identified. Finally the precise mechanism as to how amidation is activated in the peritoneum was not defined. Further studies are required.

In conclusion, PMCs express AM and amidation activity is high in the peritoneal effluent of PD patients.
